# How Does Gender Affect Leadership Communication and Job Satisfaction Among Physicians? Evidence from Swiss Hospitals

**DOI:** 10.2147/JHL.S521242

**Published:** 2025-08-30

**Authors:** Sabina C Heuss, Tsvetana Spasova, Juliane Felder, Souvik Datta

**Affiliations:** 1 School of Business, University of Applied Sciences and Arts Northwestern Switzerland FHNW, Olten, Switzerland; 2 Centre for Energy Policy and Economics, ETH Zürich, Zürich, Switzerland

**Keywords:** leadership communication, gender, healthcare communication, team communication, physician, hospital, healthcare, generation

## Abstract

**Purpose:**

Studies show that women communicate and perceive communication from supervisors and colleagues differently than men. This is evident also in the healthcare sector and particularly among female doctors. The primary aim of this study is to explore the relationships between communication practices, leadership approaches, and the association with physicians’ job satisfaction and overall well-being. Particular attention is paid to gender and its impact on the communication of physicians in hospitals.

**Design/Methodology/Approach:**

We carried out a comprehensive survey of physicians working in hospitals across Switzerland in 2019 to investigate the role of gender on communication style and physicians’ job satisfaction and well-being in hospitals. We collected 1565 responses and performed Mann–Whitney *U*-tests to test if the job satisfaction and well-being measures differ by gender. Furthermore, we used multiple-regression models to estimate the conditional relationship between the outcome measures and predictor variables.

**Findings:**

Effective leadership communication is positively associated with the job satisfaction of all physicians, regardless of functions, generations, languages, genders, hospital types, and specializations. The results of the study indicate that there are gender differences between men and women physicians in Swiss hospitals regarding the effect of leadership communication on job satisfaction, satisfaction with leadership communication and well-being.

**Originality:**

While there are valid studies that provide valuable insights into leadership styles, gender disparities in leadership, and the impact of gender bias on leadership, this study fills the lack of direct evidence addressing the effect of leadership communication skills on the job satisfaction and the well-being of female physicians and the need for different types of leadership communication skills for female physicians in hospitals.

## Introduction

Communication is the most important tool in leadership.^1^ As Scott, Mitchell^2^ noted, “Communication is the critical process in organizing because it is the primary medium of human interaction”. There are various components that can influence the leadership task and the outcome of leadership, namely the performance of employees.^3^ One important component is gender. The impact of gender on communication skills and communication styles as well as the perception of communication has been subject of extensive research.^4–7^ The literature suggests that gender significantly influences perceptions of leadership effectiveness, and this idea is reinforced by the assumption that women as a group display higher levels of social and emotional skills.^8–10^ The results seem to indicate, not only in Switzerland, that women in management positions and their management communication are perceived differently, even if they have the same patient outcomes as male doctors^11^,^12^ and face various gender-specific multifactorial disadvantages.^13^ Female physicians often face prejudicial evaluations of their competence, which can undermine their effectiveness and influence within healthcare teams.^14^,^15^ These results show that women in management positions in hospitals not only communicate differently but are also perceived differently. These differences in perception are reinforced by the traditionally strong hierarchical management structures in hospitals worldwide, where the focus is on the head physician. This position is still largely held by a man.^16–18^

In a previous article Heuss, Datta^19^ have already shown that certain leadership communication skills, information quality and amount of feedback correlate positively with job satisfaction and well-being of physicians in Swiss hospitals. The findings of the analysis for this article indicate that various aspects of leadership communication—such as tone, the quality of the information shared, communicative behavior, and the provision of feedback—are key contributors to enhancing job satisfaction and employee well-being. These factors appear to exert a particularly strong influence on the well-being of younger physicians, especially those from Generation Y (born post-1980). Among medical professionals, residents experience the most pronounced positive effects of leadership communication on job satisfaction, whereas this influence diminishes at higher levels of the organizational hierarchy.^19^ While there are valid studies that provide valuable insights into leadership styles, gender disparities in leadership, and the impact of gender bias on leadership,^20–24^ there is a lack of direct evidence addressing the effects of leadership communication skills on the job satisfaction and the well-being of female physicians and the need for different types of leadership communication skills for female physicians in hospitals.

The study presented aims to evaluate how gender is associated with various facets of leadership communication, including tone, information quality, and feedback, job satisfaction and well-being of physicians employed in Swiss hospitals. While the multivariate analyses that were conducted provided different insights into leadership communication’s correlation with factors such as generational affiliation, functional affiliation, language, hospital type, and medical specialty,^19^ this paper focuses on the effects of gender on the communication between physicians and on the leadership communication skills of superiors with their team members in hospitals.

To be able to categorize the results of the study, it is crucial to examine the concepts of leadership and communication. Numerous definitions exist, with Hackman, Johnson^25^ characterizing it as “human symbolic communication that modifies the attitudes and behaviors of others to achieve shared group goals and needs”. This definition highlights the inseparable nature of leadership and communication, as effective communication skills are essential for realizing leadership objectives.^1^ Consequently, communication is regarded as a potent tool wielded by leaders,^26^ aimed at imparting knowledge, planning, coordinating, and evaluating.^27^

The concept of communication competence as a facet of leadership competencies has been investigated in various studies, yielding diverse definitions of leadership communication, dating back two decades.^28–31^ Recurring elements can be identified, as summarized by Hertzsch, Schneider, Maier,^28^ encompassing behavior, cognition, motivation, and notably, the situational and social appropriateness of communication, as described by Six, Gimmler.^32^ These findings are corroborated by a literature review in the healthcare sector by Martins, De Sousa, Abrantes, da Silva Pinto, de Almeida Gomes, Martins, Coutinho, Negrão, Baptista, Fernandes,^33^ examining the relationship between communication and leadership specifically in healthcare. The review elucidates the link between communication and leadership in healthcare, highlighting that effective leadership hinges on proficient communication to guide and direct, while communication is one of the most crucial leadership skills.^34^

Communication proficiency of leaders is also the most pivotal attribute influencing employee satisfaction.^35–37^ A literature review by Litmanen^38^ indicated that leadership communication styles exert varying influences on employee well-being, evident in performance and behavior. Positive impacts of leadership communication skills in healthcare have also been observed with regard to team motivation, patient care, and patient safety^39^ as well as well-being and job satisfaction of physicians.^40^

## Materials and Methods

We developed a questionnaire (for more details on methods such as sample size, time of study and more, see Heuss, Datta)^19^ and sent it to physicians in hospitals across Switzerland (see full questionnaire in appendix). The sample consists of physicians in Swiss hospitals in all parts of the country and language regions. Data was collected between October 01, 2019, and November 30, 2019, and 1565 individuals responded.

The concepts underlying the questions in our survey are given in Table 1. This article examines communication practices, leadership approaches, and their effects on physicians’ job satisfaction and well-being, with a focus on gender in hospital communication. Therefore, the human value scale and parts of other concepts are not included in the scope of this article.

**Table 1:** Overview of Leadership Communication Concepts

**Leadership Communication**
Feedback
I would like more active feedback from direct superior (DS).
I often receive spontaneous feedback from DS.
I receive regular standardized feedback from DS.
I receive regular clear target agreements from DS.
I receive motivating praise from DS.
I receive frustrating criticism from DS.
I discuss long-term career goals with DS.
Tone of Communication
Friendly
Appreciative
Transparent
Helpful
Equal (non-discriminatory)
Not hardened
Information quality and information behavior
Sincere
Proactive
Trust-building
Helpful
**Leader-Member-Exchange (LMX)**
… my superior would use his/her influence to help me with work problems.
… makes an effort to accommodate my work-life balance.
… is good at assessing how many tasks I can complete.
… allows me to learn a lot of content.
… enables me to learn a lot about leadership.
… is transparent in his/her assessment of me.
… recognizes my development opportunities.
**Well-being**
I did not have enough time to complete tasks carefully.
My work was emotionally demanding.
I withheld my opinion for fear of negative consequences.
I withheld my opinion because it was not being considered.
My work demanded so much energy that it negatively affected my personal life.
I had problems relaxing.
**Stabilized work satisfaction versus resigned work dissatisfaction**
I am satisfied overall and hope that my work situation will remain as it is in the future.
I am satisfied overall but would like my work situation to improve further.
I am not satisfied with some things about my work situation, but I believe I can make improvements.
I am satisfied overall because I do not think you should expect too much from your work situation.
I am not satisfied with my work situation, and I do not think anything can be improved about it.

The concepts that were queried for this study have been tested and used several times in different settings and contexts. The model by Bruggemann^41^ examined job satisfaction, which was divided into five types: stabilized work satisfaction, constructive work satisfaction, constructive work dissatisfaction, resigned work satisfaction, and resigned work dissatisfaction.^42^ A questionnaire based on the third version of the Copenhagen Psychosocial Questionnaire (COPSOQ III) was used to assess well-being.^43^,^44^ The topic of feedback is partly based on the HR Barometer^45^ and modified to fit the specific objectives of this survey. One section of the survey focused on the amount and type of feedback physicians received, their expectations regarding feedback, and the sources from whom they received it. Another section of the survey relevant to the current article explored the tone and quality of communication, and the questions were developed specifically for this study. The question was: “How would you describe the tone of communication between you and your superiors?” The possible answers were: friendly, appreciative, transparent, helpful, equal (non-discriminatory), not hardened. The question regarding information quality and information behavior quality was formulated as follows: How do you feel about the information behavior of your superiors? The possible answers were sincere, proactive, trust-building and helpful. These items were created by the scientific advisory council overseeing the study and were tested and assessed as outlined in the article by Heuss, Datta.^19^

The questionnaire on perceived leadership communication arose from the desire to research communication and leadership qualities in a theoretically sound manner and to be able to survey them in a questionnaire. Many tried and tested questionnaires on various aspects of leadership and leadership styles were used for this purpose (Schneider, Maier, Lovrekovic, and Retzbach, 2015). The underlying model by Hertzsch, Schneider, and Maier examines interpersonal communication in organizations and enables a differentiated view of leadership behaviour in a wide variety of communication situations. Communication deficits and problems can be identified through the view of dyadic leadership communication by both the supervisor and the corresponding perception by the employee.

The notion of Leader-Member-Exchange (LMX) originated with research conducted by Graen.^46^ Graen defined the responsibilities and functions of workers much beyond what is stated in the employment contract and where workers are offered additional responsibilities or roles, which they might accept or decline (role creation). This may result in role routinization, which Graen, Scandura^47^ define as a “high-quality” and consistent working relationship between managers and staff. This dyadic process’s quality is measured by the Leader-Member-Exchange Scale, which has seven components. The aim of the questionnaire is to know the level of confidence, respect and trust and questions about the relationship between superior and employer.^48^,^49^

An essential step in the data preprocessing involved constructing composite indices. To determine whether items from the respective questionnaires could be aggregated into coherent indices, we employed principal component analysis alongside internal consistency assessments (such as reliability analysis). Given the distributional properties of the data—including deviations from normality, the presence of numerous outliers, and the comparison of more than two groups—we conducted univariate group comparisons using the nonparametric Mann-Whitney *U*-test. This omnibus test is appropriate for detecting group-level differences when parametric assumptions are violated.

Apart from the univariate tests, we also modelled the outcomes using multiple linear regression. The variables that we used to identify the variation in the outcome variables are socio-demographic variables like age, language used in answering the questionnaire (German, French or Italian), the specialty of the doctor (internal medicine, surgeon or psychiatrist), and the type of hospital (regional, cantonal, university, psychiatric, rehabilitation clinic and private).

## Results

The evaluations show clear differences in the effects of gender on the various categories of leadership communication in the hospital. In the following, the parts where significant differences were found will be discussed in detail. These are the gender differences in the Leader-Member-Exchange concept, in the tone of communication, in information behavior and in feedback. The Leader-Member-Exchange concept can show how well line managers and employees know, appreciate, respect and trust each other.

### Bivariate Evaluations

#### Effects of Gender on Physician’s Job Satisfaction and Well-Being

The correlation of leadership communication with well-being and job satisfaction was examined in the aggregate. Job satisfaction is highly influenced by the quality of the information. Higher work satisfaction is correlated with higher-quality information across all generations and function levels. Compared to their more senior colleagues, residents and senior physicians had lower opinions on the quality of the information. The word “sincere” is what physicians mention the most. A good tone of communication has a greater impact on job satisfaction among female physicians than among male physicians. It shows clearly in the bivariate correlation that the tone of information (alongside feedback and quality of information) has the greatest influence on job satisfaction, but also on the well-being of doctors. This effect is more pronounced among women, as Table 2 below illustrates. In addition, women react more positively to superiors and chief physicians who are sensitive, seek to discuss problems more often and deal constructively with their own mistakes.

**Table 2:** Bivariate Correlations: Dimensions of Job Satisfaction and Dimensions of Leadership Communication Between Genders

		PLC Direct superior	PLC Head physician	Tone of communication	Information quality
Female physicians	Stabilized job satisfaction	0.51*	0.51*	0.58*	0.57*
	Resigned job satisfaction	0.34*	0.33*	0.38*	0.40*
Male physicians	Stabilized job satisfaction	0.30*	0.20*	0.38*	0.40*
	Resigned job satisfaction	0.36*	0.24*	0.41*	0.31*

**Note**: *p < 0.05.

### Multivariate Evaluations

#### Well-Being and Gender

The well-being of women is significantly worse than that of their male colleagues, as shown below (see Table 3). The reliability of the items used to calculate the well-being index, as measured by Cronbach’s alpha, is 0.75. The survey asked whether physicians had too little time to prepare their tasks, withheld their opinion for fear of consequences or whether their work had a negative impact on their private life.

**Table 3:** Regression Model of Index Well-Being

	1 (All)	2 (Resident Physicians)	3 (Senior Physicians)	4 (Attending Physicians)	5 (Head Physicians)
Female	−0.120** (−3.12)	−0.138* (−1.86)	−0.0912 (−1.46)	−0.0810 (−1.00)	−0.178 (−1.53)
Senior physicians	0.0655 (1.23)				
Attending physicians	−0.0730 (−1.04)				
Intercept	2.918** (29.25)	3.081** (12.36)	2.960** (16.07)	2.964** (11.11)	2.429** (6.03)
Observations	1488	407	526	335	220

**Notes**: t statistics in parentheses. *p < 0.10, **p < 0.05.

In all items, women agreed more strongly with the negative impact on their private life than men. Overall, women are more likely to have problems relaxing, are more likely to experience negative consequences of the workload on their private lives, are more likely to hold back their opinions, find work more emotionally demanding and consider the time available to be too little to complete tasks carefully.

We performed a Mann–Whitney *U*-test to evaluate whether the well-being index differed by gender. The results indicated that females had significantly lower values than males (z = −3.44, p = 0.001), indicating that the Index Well-being is, on average, worse compared to males. A detailed regression analysis of the variable Index Well-Being, shown in Table 3, indicates that female physicians have a lower index (by around 0.12) compared to males and this is statistically significant (column 1). This effect remains significant for resident physicians (column 2) while for the other three groups (columns 3, 4, and 5), the impact of gender is not significant. We performed a check for multicollinearity and the VIFs were calculated as follows: the maximum VIFs were 2.72, 2.57, 2.70, 2.26, and 3.95 for the columns (1), (2), (3), (4), and (5), respectively. The mean VIFs were less than 2 for all columns. These values indicate that multicollinearity is not an issue.

#### The Effects of Gender on Tone of Communication

The tone of communication was queried with the comparisons friendly - no friendly, appreciative – not appreciative, transparent – not transparent, helpful- not helpful, discriminatory – not-discriminatory, hardened – not hardened. Overall, the results show that physicians in Swiss hospitals have a pleasant tone of communication and high-quality information behaviour. Physicians are rating the communication tone in general as “friendly” (see Table 1: Overview of Leadership Communication Concepts). The reliability of the items used to calculate the tone of communication index, as measured by Cronbach’s alpha, is 0.94. The tone of communication was labelled less frequently as “helpful” and “transparent”. The tone of communication is typically seen as better than the information quality (sincere, pro-active, trust-building, helpful). Physicians in general describe the information quality and the actions of their superiors as “sincere” but not as “proactive”.

We performed a Mann–Whitney *U*-test to evaluate whether the tone of communication, as measured by the variable “Index Tone of Communication” (see Table 1), differed by gender. The results indicated that females had significantly lower values of the index than males (z = −2.95, p = 0.003), hence female physicians perceive the tone of communication with their peers to be significantly more negative than that of their male counterparts. This also applies to female physicians’ perceptions of the behaviour and quality of information (see below).

We performed a more detailed regression analysis to evaluate the association between gender and the tone of communication index and report the results in Table 4. The columns refer to different groups of physicians. Column (1) takes all the physician groups together, column (2) is for the group resident physicians, column (3) for the group senior physicians, column (4) for the group attending physicians, and column (5) for the group head physicians. The tone of communication index was lower, on average, for females compared to males by around 0.2 units. We found that the impact of gender is statistically significant for senior and head physicians with females having a lower index compared to males, though the effect is only significant at the 10% level for head physicians. A check for multicollinearity indicates that there is none. The maximum VIFs were 2.72, 2.57, 2.70, 2.25, and 3.95 for the columns (1), (2), (3), (4), and (5), respectively. The mean VIFs were less than 2 for all columns.

**Table 4:** Regression Model of Tone of Communication Index

	1 (All)	2 (Resident Physicians)	3 (Senior Physicians)	4 (Attending Physicians)	5 (Head Physicians)
Female	−0.204** (−2.70)	−0.149 (−1.06)	−0.289** (−2.35)	0.00670 (0.04)	−0.465* (−1.91)
Senior physicians	0.286** (2.72)				
Attending physicians	0.401** (2.90)				
Head physicians	0.274* (1.71)				
Intercept	6.133** (31.11)	5.531** (11.74)	6.331** (17.32)	7.115** (13.57)	6.385** (7.68)
Observations	1489	407	525	336	221

**Notes**: t statistics in parentheses. *p < 0.10, **p < 0.05.

#### The Effects of Gender on Feedback

The feedback item was surveyed in three dimensions. Firstly, physicians were asked how much feedback they would like. Secondly, they were asked how much feedback they receive from their superiors. And thirdly, physicians were asked how much feedback they give to their employees. To evaluate feedback, the questionnaire used an index built on these questions and divide per “my direct superior (DS)” and per “my head physician” (see Table 1). The reliability measures, as indicated by Cronbach’s alpha, are 0.80 and 0.94 for the two feedback variables, respectively.

We performed a Mann–Whitney *U*-test to evaluate whether the desirability of active feedback from direct the superior differed by gender. The results indicated that females had significantly higher values than males (z = 5.06, p < 0.01), indicating that females, in general, desire more active feedback from their direct superior compared to males.

Again, we performed a more detailed regression analysis to evaluate the association between gender and the desirability of active feedback from the direct superior and report the results in Table 5. We found that the impact of gender is statistically significant only for resident physicians (2) with females desiring more active feedback (by 0.3) compared to males. This effect is not significant for either senior, attending, or head physicians (columns (3), (4), and (5)). A check for multicollinearity indicates that there is none. The maximum VIFs were 2.84, 2.57, 2.70, 2.26, and 3.98 for the columns (1), (2), (3), (4), and (5), respectively. The mean VIFs were less than 2 for all columns.

**Table 5:** Regression Model of Feedback by Direct Superior

	1 (All)	2 (Resident Physicians)	3 (Senior Physicians)	4 (Attending Physicians)	5 (Head Physicians)
Female	0.177** (2.13)	0.300** (2.26)	−0.0221 (−0.14)	0.270 (1.52)	0.135 (0.52)
Senior physicians	−0.0814 (−0.69)				
Attending physicians	−0.381** (−2.53)				
Head physicians	−0.148 (−0.84)				
Intercept	5.437** (24.55)	5.833** (13.05)	5.519** (11.14)	4.856** (8.35)	4.808** (5.39)
Observations	1286	406	336	330	215

**Notes**: t statistics in parentheses. **p < 0.05.

There are also differences in the desire for active feedback. Who gives the feedback is relevant here. Women express a higher desire for feedback from the head physician.

We performed a Mann–Whitney *U*-test to evaluate whether the perception of receiving active feedback from the head physician differed by gender. The results indicated that females had significantly lower values than males (z = −2.51, p = 0.012), indicating that females, in general, have the perception of receiving less active feedback from their head physician compared to males.

Again, we performed a more detailed regression analysis and report the results in Table 6. We found that the impact of gender is statistically significant for both resident and senior physicians with females receiving less active feedback, by around 0.2 for resident physicians (column (1) in Table 6) and 0.25 for senior physicians (column (2) in Table 6), compared to males. A check for multicollinearity indicates that there is none. The maximum VIFs were 2.47 and 2.84 for the columns (1) and (2), respectively. The mean VIFs were less than 2 for all columns.

**Table 6:** Regression Model of Feedback From the Head Physician

	1 (Resident Physicians)	2 (Senior Physicians)
Female	−0.196* (−1.79)	−0.246** (−2.59)
Intercept	3.594** (9.75)	4.546** (15.92)
Observations	402	496

**Notes**: t statistics in parentheses. *p < 0.10, **p < 0.05.

#### The Effects of Gender on Information Quality

Information quality was indexed within the questionnaire with the following question: How is your perception of the information quality of your superiors? And the following possible answers: sincere, proactive, confidence-building, helpful (see Table 1: Overview of Leadership Communication Concepts). The reliability measure, as indicated by Cronbach’s alpha, is 0.59 for the information quality index variable. A Mann–Whitney *U*-test to evaluate whether the index for information quality differed by gender showed that females had significantly lower values than males (z = −2.53, p = 0.011), indicating that the perception of females of the information quality of their superiors is, in general, worse compared to males. However, a more detailed regression analysis showed that, after controlling for other characteristics, the impact of gender becomes insignificant in the different physician groups (see columns (2), (3), and (4) in Table 7) but, if we take all the groups together, the effect is statistically significant (see column (1) in Table 7). There may be various reasons why the individual regressions do not show a significant effect even though the coefficients show a consistently negative value. It could be that there is too much variation. Further research is needed to investigate this in more detail.

**Table 7:** Regression Model of Information Quality

	1 (All)	2 (Resident Physicians)	3 (Senior Physicians)	4 (Attending Physicians)	5 (Head Physicians)
Female	−0.223** (−2.58)	−0.214 (−1.37)	−0.227 (−1.58)	−0.125 (−0.67)	−0.413 (−1.51)
Senior physicians	0.150 (1.25)				
Attending physicians	0.305* (1.94)				
Head physicians	0.0952 (0.52)				
Intercept	5.797** (25.74)	5.262** (10.02)	5.964** (13.95)	6.701** (11.02)	5.632** (6.03)
Observations	1489	408	525	335	221

**Notes**: t statistics in parentheses. *p < 0.10, **p < 0.05.

#### The Effects of Gender on Perceived Leadership Communication

A Mann–Whitney *U*-test to evaluate whether the index for perceived leadership communication differed by gender indicated that females had significantly lower values than males (z = −2.75, p = 0.006), indicating that the perception of females of perceived leadership communication is, in general, worse compared to males. The reliability measure, as indicated by Cronbach’s alpha, is 0.59.

A subsequent regression analysis showed that men and women differ in their perception of leadership communication as well as in their self-perception (see Table 8). A check for multicollinearity indicates that there is none. The maximum VIFs were 2.81, 2.57, 2.65, 2.26, and 3.78 for the columns (1), (2), (3), (4), and (5), respectively. The mean VIFs were less than 2 for all columns. A further Mann–Whitney *U*-test indicated that women rate themselves more critically than men in terms of sensitivity, dealing constructively with criticism and proactively initiating discussions when problems arise (z = −3.506, p = 0.0005).

**Table 8:** Regression Model of Self-Perception of Leadership Communication

	(1) All	(2) Resident Physicians	(3) Senior Physicians	(4) Attending Physicians	(5) Head Physicians
Female	−0.0724** (−1.97)	−0.170** (−2.22)	−0.0367 (−0.60)	0.00649 (0.09)	−0.00590 (−0.07)
Senior physicians	0.500** (9.79)				
Attending physicians	0.444** (6.63)				
Head physicians	0.429** (5.52)				
Intercept	4.574** (47.40)	4.533** (17.71)	5.058** (27.99)	5.122** (21.46)	4.864** (15.63)
Observations	1366	409	404	333	220

**Notes**: t statistics in parentheses. **p < 0.05.

On the other hand, women are highly significantly more critical in their assessment of direct superiors than men (See Table 9). A check for multicollinearity indicates that there is none. The maximum VIFs were 2.60, 2.57, 2.85, and 2.26 for the columns (1), (2), (3), and (4), respectively. The mean VIFs were less than 2 for all models. The reliability measure, as measured by Cronbach’s alpha, is 0.82 for the index. The assessment of colleagues is more negative than the self-perception. It also emerges that those who are most critical of themselves are also more critical of others.

**Table 9:** Regression Model of Perceived Leadership Communication

	(1) All Except Head Physicians	(2) Resident Physicians	(3) Senior Physicians	(4) Attending Physicians
Female	−0.130** (−2.24)	−0.143 (−1.56)	−0.191* (−1.82)	−0.0368 (−0.38)
Senior physicians	−0.0783 (−1.01)			
Attending physicians	0.0885 (0.84)			
Intercept	4.709** (30.67)	4.288** (13.94)	4.860** (15.36)	4.938** (15.45)
Observations	1229	409	490	330

**Notes**: *t* statistics in parentheses. *p < 0.10, **p < 0.05.

#### The Effects of Gender on Leader-Member-Exchange

The evaluation shows that women are more critical than men in their assessment of the confidence, respect and trust in their superiors, as was measured with the Leader-Member-Exchange model.^48^ Women are less likely to assume that head physicians would use their influence to help them or take their work-life balance into consideration. Once more, female doctors and younger doctors (Generation Y) are more critical in their assessment of the management style and communication of superiors (see Table 10). A check for multicollinearity indicates that there is none. The maximum VIFs were 2.83, 2.37, 2.67, and 2.26 for the columns (1), (2), (3), and (4), respectively. The mean VIFs were less than 2 for all models. The reliability of this index, according to Cronbach’s alpha, is 0.90.

**Table 10:** Regression Model of Leader-Member-Exchange

	(1) All Except Head Physicians	(2) Resident Physicians	(3) Senior Physicians	(4) Attending Physicians
Female	−0.109* (−1.72)	−0.167 (−1.51)	−0.260** (−2.63)	0.215* (1.74)
Senior physicians	0.305** (3.58)			
Attending physicians	0.548** (4.76)			
Intercept	4.540** (26.96)	4.002** (10.77)	5.275** (17.67)	4.811** (11.82)
Observations	1231	399	500	332

**Notes**: t statistics in parentheses. *p < 0.10, **p < 0.05.

## Discussion

This study provides an analysis of gender differences in leadership communication among physicians in Swiss hospitals, highlighting some significant disparities in several key areas. The findings reveal that female physicians consistently report a more negative perception of communication dynamics, particularly in terms of communication tone, feedback, and information quality, compared to their male counterparts. Specifically, female physicians rate the tone of communication as less friendly and helpful, and they perceive their superiors’ information behavior as less proactive and sincere. This trend is particularly pronounced among senior and head physicians.

Additionally, gender differences emerge in the desire for feedback. Female physicians generally express a greater need for feedback from their direct superiors and show more interest in receiving feedback from head physicians compared to male physicians. These gendered perceptions of feedback are not only linked to communication but also to broader leadership dynamics, as evidenced by the Leader-Member-Exchange (LMX) model. Female physicians tend to assess the trust and respect from their superiors more critically than their male peers, which could reflect broader patterns of gendered experiences in workplace leadership and mentorship. Nonetheless, the available data does not reveal the information that women receive less favorable treatment or lower-quality information compared to men.

The results indicate that female physicians tend to be more self-critical in evaluating their communication skills compared to their male counterparts. Additionally, they are more critical of their colleagues, particularly residents. In contrast, male physicians generally perceive both themselves and their colleagues in a less critical light.

Moreover, the study identifies a significant link between leadership communication and job satisfaction, particularly among female physicians. A positive tone, transparent communication, and constructive feedback are more influential in enhancing the job satisfaction and well-being of female physicians compared to their male counterparts. Good leadership communication as well as a good tone of communication and information quality have a greater impact on job satisfaction among female physicians than among male physicians. Female physicians report lower levels of well-being and job satisfaction and are more likely to experience negative impacts of work-life balance.

The data show no significance in the correlation between information quality and gender. There may be various reasons for this. The figures do show a consistent pattern. However, too many variations can lead to this result. Further research is needed to investigate this result in more detail.

Comparisons with research literature on the influence of gender on leadership communication show that language and communication is an important indicator of identity and social interaction and is strongly dependent on gender and gender patterns.^50–52^ In communication, equality and social interaction seem to be more important to women than to men, whose communication patterns are more likely to be characterized by the keywords control, setting guidelines and conversational dominance.^53^ This could therefore be an explanation for the results of the present study. If women’s communication is more focused on the inclusion of all interlocutors, on the relationship with them and on the joint achievement of a goal, the focus on control, specifications and self-expression of the male-connoted communication style could explain negative effects on women in hospital.

In addition, there are numerous studies on the different value and evaluation systems by which men and women must measure themselves and be measured in communication.^54^ Organizational cultures and the specific circumstances in the healthcare sector also led to gender differences being reinforced.^55–57^

Gender as a category of analysis must be understood within a broader nexus of intersecting social variables, including ethnicity, age, educational attainment, socioeconomic background, emotional states, and the power asymmetries embedded in specific communicative contexts such as in the healthcare setting.^54^ Contemporary research increasingly emphasizes the dynamic co-construction of identity in discourse, where gender represents only one of several interacting dimensions.^55^ Accordingly, investigations into gendered communication must account for the entire interactional and situational framework. While biological sex remains a component of categorization, it is not necessarily the only analytically salient one in every context. Professional and institutional roles, cultural affiliations, and ethnic backgrounds often exert greater influence in shaping communicative patterns.^54^

Gender never operates in isolation but always in tandem with other social determinants. This conceptual framework is captured by the notion of intersectionality.^56^ However, due to the intricate entanglement of these factors, it is generally untenable to isolate one as universally dominant. Only nuanced, qualitative inquiry can adequately illuminate the layered complexities inherent in such communicative phenomena.^54^

Although the findings reveal statistically significant associations—particularly among female physicians—between leadership communication (eg, tone or feedback) and reported job satisfaction, these relationships may be bidirectional. It is plausible that higher job satisfaction leads to more favorable perceptions of leadership communication, rather than (or in addition to) communication driving satisfaction. There is a need for longitudinal studies or experimental interventions to better understand the directionality and potential mediating mechanisms between leadership communication, job satisfaction and well-being.

## Limitations

All surveys have several known limitations, some of which are mentioned here. The survey sample does not include those who did not respond, so response bias may occur. This can affect the results because answers are influenced by this bias. Response bias can also occur when respondents answer as the researcher expects. Since our survey was voluntary and not everyone responded, there may be selection biases. We have performed some comparisons of our sample with the available data from the Swiss hospital sector and, based on some observables, our sample is quite similar to the general features of doctors who work in Swiss hospitals.^57^ The percentage of women in our sample is 49%, while published data from FMH, the Professional Association of Swiss Physicians, reports 47% women who worked in the hospital sector in 2019. The average age of doctors is also similar with 43 years in our sample and 44 years reported by FMH, the Professional Association of Swiss Physicians. The percentage of doctors with a non-Swiss degree is 36%, which is close to the 40.2% reported by the FMH. Therefore, while we cannot ensure a complete match due to non-observables, it is reassuring that the observable demographics in our sample are similar to the sector in general. Also, our findings can only establish an association between the outcome variables and predictors and cannot make any claims about the direction of causality in our results. Furthermore, it should be noted that the survey only refers to doctors in Swiss hospitals.

## Conclusion

The results of the study indicate that there are gender differences between men and women physicians in Swiss hospitals. Effective leadership communication has a positive association with the job satisfaction of all physicians, regardless of functions, generations, languages, genders, hospital types, and specializations. This was seen and demonstrated in previous evaluations that looked at leadership style, feedback-giving, and the quality and behaviour of information.^19^ Regarding the impact of positive leadership communication on well-being and work satisfaction, the findings are rather consistent.

The study highlights the complex relationship between gender and leadership communication, with important implications for improving organizational practices in healthcare settings. These findings suggest that addressing gender disparities in communication, feedback, and leadership practices could enhance the work environment and well-being for all physicians, but especially for women. Female physicians consistently report lower satisfaction with feedback, tone, and information quality from their superiors and are more self-critical in their assessments. These disparities are particularly pronounced among younger generations. Efforts to foster more supportive and transparent communication cultures, along with targeted strategies to improve work-life balance, are essential steps in promoting gender equity in healthcare leadership.

For hospital management, these findings underscore the need for targeted interventions to improve leadership communication. Practical steps that could directly address the results of the present study could be: a) Implementing structured feedback mechanisms that ensure regular, constructive, and transparent communication across all hierarchical levels and among all genders, b) Training hospital leaders in leadership communication, focusing on tone, responsiveness, and clarity, c) Promoting inclusive leadership development programs that actively support the advancement of women into senior roles and d) Monitoring the communication climate regularly to identify and address emerging disparities.

As is assumed the proportion of women in medicine will continue to grow and female physicians in management positions in hospitals will increase. These female leaders will influence communication, not just be influenced by it. There is a need for further research specifically focused on the effect of leadership communication skills on the well-being and job satisfaction of female physicians and the potential need for tailored leadership communication for female physicians in hospitals.
